# Prostate Cancer and the Mevalonate Pathway

**DOI:** 10.3390/ijms25042152

**Published:** 2024-02-10

**Authors:** Patricia Guerrero-Ochoa, Sergio Rodríguez-Zapater, Alberto Anel, Luis Mariano Esteban, Alejandro Camón-Fernández, Raquel Espilez-Ortiz, María Jesús Gil-Sanz, Ángel Borque-Fernando

**Affiliations:** 1 Health Research Institute of Aragon Foundation, 50009 Zaragoza, Spain; pguerrero@iisaragon.es (P.G.-O.); acamon@iisaragon.es (A.C.-F.); respilez@salud.aragon.es (R.E.-O.); mjgilsa@salud.aragon.es (M.J.G.-S.); 2 Minimally Invasive Research Group (GITMI), Faculty of Veterinary Medicine, University of Zaragoza, 50009 Zaragoza, Spain; rzapater@unizar.es; 3 Department of Biochemistry and Molecular and Cellular Biology, Faculty of Sciences, University of Zaragoza, 50009 Zaragoza, Spain; anel@unizar.es; 4 Department of Applied Mathematics, Escuela Universitaria Politécnica de La Almunia, Institute for Biocomputation and Physic of Complex Systems, Universidad de Zaragoza, 50100 La Almunia de Doña Godina, Spain; 5 Department of Urology, Miguel Servet University Hospital, 50009 Zaragoza, Spain; 6 Area of Urology, Department of Surgery, Faculty of Medicine, University of Zaragoza, 50009 Zaragoza, Spain

**Keywords:** prostate cancer, cholesterol, statins, mevalonate pathway, antitumoral

## Abstract

Antineoplastic therapies for prostate cancer (PCa) have traditionally centered around the androgen receptor (AR) pathway, which has demonstrated a significant role in oncogenesis. Nevertheless, it is becoming progressively apparent that therapeutic strategies must diversify their focus due to the emergence of resistance mechanisms that the tumor employs when subjected to monomolecular treatments. This review illustrates how the dysregulation of the lipid metabolic pathway constitutes a survival strategy adopted by tumors to evade eradication efforts. Integrating this aspect into oncological management could prove valuable in combating PCa.

## 1. Introduction

The centrality of AR in orchestrating metabolic reprogramming for the growth and advancement of PCa has been established in recent research [1]. Moreover, it has been demonstrated that the interplay between AR and the *serum response element (SRE)* gene regulatory sequence, along with other non-traditional pathways, assumes crucial significance in the context of castration-resistant PCa (CRPC). Significantly, a substantial body of research is currently focusing on this particular domain. In this context, cholesterol is one of the lipids that has great relevance due to its role in the structure of the cell membrane as well as in signaling pathways linked to the AR pathway, not only due to its precursor role in the generation of androgens, which are the main AR ligands currently used as a target for therapies. While fundamental evidence underscores the profound influence of lipids and, specifically, cholesterol on tumors, the clinical sphere remains marked by conflicting outcomes. Due to this strong evidence, several clinical trials using statins have been launched, and these trials have confirmed the important roles of cholesterol and drugs designed to lower its serum levels in the treatment of PCa.

The current research initiatives are prominently centered around delineating the lipidome, transcriptomics, and metabolomics, entailing a comprehensive quantitative assessment of lipids and metabolites through the application of liquid or gas chromatography coupled with mass spectrometry. These efforts are directed towards exploring their implications across various disease spectrums, encompassing aging and cancer, as highlighted by recent findings [2]. Over the past decade, compelling evidence has emerged, showcasing distinct lipid species originating from diverse biological sources that distinguish PCa patients from controls [3,4,5].

## 2. The Mevalonate Pathway

De novo cholesterol generation is facilitated by the utilization of glucose, glutamine, and acetate as nutrients which contribute to the production of acetyl-coenzyme A (acetyl-CoA), a primary substrate for the mevalonate (MVA) pathway [6,7,8]. However, the major source of acetyl-CoA originates from fatty acids (FAs) [4], whose synthesis, uptake, β-oxidation, and accumulation are upregulated to support the enhanced cellular proliferation and heightened demands for plasma membrane synthesis and energy production in cancer cells [9]. Intracellular lipid accumulation in lipid droplets and tumor-associated adipocytes facilitates survival under both normoxic and hypoxic conditions [10,11]. Lipid droplets play a crucial role in membrane remodeling and composition through the trafficking of fatty acids among different lipid classes stored in diverse lipid pools and organelles [12]. Notably, the prominent fatty acid transporter CD36 is frequently gained or amplified in PCa, correlating with poor prognoses [13].

Acetyl-CoA emerges as the product of enzymatic activity orchestrated by acetyl-CoA synthase short-chain family member (ACSS), ATP-citrate lyase (ACLY), and the pyruvate dehydrogenase complex (PDC) [14]. Notably, these metabolic enzymes translocate to the nucleus, collaborating with diverse transcriptional factors and regulators to modulate gene expression through the acetylation of histones [15,16,17]. The orchestration of DNA methylation, histone posttranslational modifications (PTMs), and noncoding RNAs (ncRNAs) significantly impacts gene expression in PCa, contributing to tumorigenesis and its progression [18]. Within the context of PCa, a nuclear subunit of PDC, pyruvate dehydrogenase A1 (PDHA1), contributes to the expression of sterol regulatory element-binding protein (SREBP)-dependent genes involved in lipid synthesis in both mitochondria and nuclei, thereby sustaining tumorigenesis [19]. Consequently, acetyl-CoA serves as the pivotal metabolite initiating the MVA pathway, which also influences histone acetylation, representing a crucial mechanism for non-mutational epigenetic reprogramming. Moreover, acetyl-CoA functions as the primary source of nicotinamide adenine dinucleotide phosphate (NADPH), the principal antioxidant in cancer cells, facilitating the management of elevated levels of reactive oxygen species (ROS) typically generated to maintain signaling and various cellular processes within tumors [20,21,22,23].

The condensation of three molecules of acetyl-CoA by 3-hydroxy-3-methylglutaryl coenzyme A (HMG-CoA) synthase sequentially generates HMG-CoA, which is then converted into MVA through the action of HMG-CoA reductase (HMG-CR). The subsequent conversion of MVA into isopentenyl diphosphate (IPP) leads to the production of vital metabolites such as farnesyl pyrophosphate (FPP), geranyl pyrophosphate (GPP), heme A, and coenzyme Q10 (ubiquinone) [24,25]. The metabolization of FPP generates lanosterol, which is further metabolized into cholesterol through an enzymatic pathway [26]. Cholesterol serves as the precursor for various crucial compounds, such as vitamin D, bile salts, oxysterols, and steroid hormones, with the latter being known to initiate and drive the progression of PCa [27].

Notably, MVA metabolites have been shown to regulate conventional and non-conventional T lymphocytes (LT) at various levels. Specifically, γδ LT recognizes lipid antigens independent of major histocompatibility complex (MHC) class I presentation, originating from the MVA pathway, with IPP being the most relevant, presented by the ubiquitous protein butyrophilin 3A1 (BTN3A1, also known as CD277), which binds to phosphorylated antigens [28]. Enzymes derived from the MVA pathway are also responsible for post-translational modifications in RAS and Rho families, essential for their functional regulation, influencing various cancer-relevant pathways, including cytoskeletal organization, vesicle trafficking, gene expression, cell signaling, cell cycle, motility, and cell survival, supporting tumor initiation, growth, metastasis, and therapy resistance [29]. The inhibition of the MVA pathway promotes cancer cell death by activating the intrinsic apoptosis pathway [30].

During the transformation of PCa cells, there is a progressive reactivation of mitochondrial oxidative phosphorylation (OXPHOS) alongside increased glucose metabolism and decreased citrate production [31,32]. Androgen signalling reprograms global cellular metabolic pathways, including aerobic glycolysis, mitochondrial respiration, and de novo lipogenesis, to meet the metabolic and biosynthetic demands of PCa cells [33,34,35]. Long-term androgen treatment also increases mitochondrial biogenesis and activity [35].

**Figure 1:**
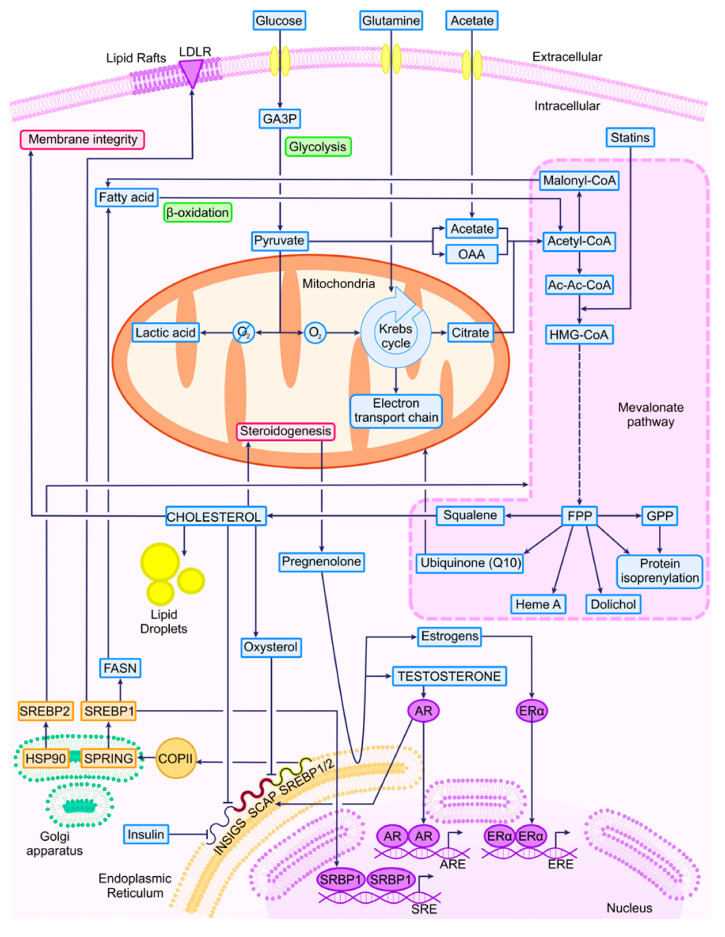
MVA pathway and related molecules. LDLR, low-density lipoprotein receptor; GA3P, glyceraldehyde 3-phosphate dehydrogenase; OAA, oxalacetate; Malonyl-CoA, malonyl coenzyme A; Acetyl-CoA, acetyl coenzyme A; Ac-Ac-CoA, aceto-acetyl coenzyme A; HMG-CoA, 3-hidroxi-3-metilglutaril-coenzima A or β-hidroxi-β-metilglutaril-coenzima A; FPP, farnesyl pyrophosphate; GPP, geranyl pyrophosphate; FASN, fatty acid synthase; SREBP, sterol regulatory element-binding protein; HSP90, heat shock protein 90; SPRING, SREBF pathway regulator in Golgi; COPII, Coat Protein Complex II; INSIGS, insulin-induced genes; SCAP, SREBP cleavage-activating protein; AR, androgen receptor; ERα, estrogen receptor α; *ARE*, *androgen response elements*; *ERE*, *estrogen response elements*; *SRE*, *sterol response elements*.

The crucial genes involved in the initiation of the MVA pathway enzymes are chiefly under the regulatory control of the SREBP family. SREBP1a and SREBP1c are transcribed from alternative start sites in the *SREBPF1* gene, with the former regulating the expression of MVA and fatty acid metabolism genes and the latter predominantly governing the expression of fatty acid metabolism genes. SREBP2, transcribed from the *SREBPF2* gene, functions as the principal transcription factor for genes associated with the MVA pathway [26,36,37]. All these considerations are shown in a schematic way in Figure 1.

## 3. Steroidogenesis and the Androgen Receptor Pathway

Under normal physiological conditions, SREBP2, in the presence of cholesterol and oxysterols, is sequestered in the endoplasmic reticulum (ER), remaining bound to the SREBP cleavage-activating protein (SCAP) complex, along with insulin-induced genes (INSIGs). Upon sterol deprivation, INSIGs undergo ubiquitination and subsequent degradation. SREBP2, along with SCAP, is then transported to the Golgi apparatus for cleavage by site-1 protease (SP1) and site-2 protease (SP2) before translocating to the nucleus to bind to *SRE* on the DNA [38,39,40].

In contrast, SREBP1 is not strongly regulated by sterol levels; rather, it is inhibited by polyunsaturated fatty acids (PUFAs) at multiple levels, including amino acids and eIF-2α kinase. It can be induced by insulin, high-glucose conditions, liver X receptors, atypical protein kinase C (PKC) isoforms, and fatty acids [40,41]. Notably, oxysterols, derivatives of cholesterol, can activate liver X receptors, which function as regulators of lipid flux and activators of SREBP1c [27].

## 4. Interrelation between Prostate Cancer, Androgen Receptor, and Cholesterol

### 4.1. The Role of Cholesterol in Plasma Membrane Lipid Rafts

The dynamic functionality of the plasma membrane is underpinned by specialized regions rich in cholesterol and sphingolipids, known as “Lipid Rafts” (LRs), which have the capacity to modulate the accessibility of proteins to regulatory or effector molecules. Cholesterol constitutes approximately 30% of the lipids in the plasma membrane and plays a pivotal role in closely packing the acyl chains of phospholipids, thereby promoting phase separation and stabilizing the formation of LRs [42]. Cancer cells exhibit higher levels of intracellular cholesterol and LRs compared to their normal counterparts [43]. For instance, in PCa, cell lines like PC-3 have been found to possess LRs at levels five times greater than those in normal cells [44].

Lipid rafts play a crucial role in sustaining proliferative signaling by hosting receptors organized within a structure referred to as CASMER, short for “cluster of apoptotic signaling molecule-enriched rafts,” where death receptors are concentrated [45,46,47]. This implies that both types of signals, those promoting cell survival and those leading to cell death, are interconnected in a dynamic manner. CASMER communicate with other subcellular structures to facilitate the transmission of apoptotic signals or induce signals related to cell survival, proliferation, inflammation, cancer growth, and metastasis. In the context of mTOR complex 1 (mTORC1) signaling, molecules like insulin-like growth factor (IGFs) and the epidermal growth factor (EGF) are involved [48].

Cholesterol is necessary to maintain lipid raft structure since the removal of cholesterol renders these structures non-functional and leads to the dissociation of proteins from the rafts [47,49]. Edelfosine, an ether-phospholipid with a high affinity for cholesterol, has emerged as an antitumor drug that acts through lipid rafts, promoting cell death by co-clustering raft-associated Fas/CD95 receptors and inducing apoptosis in a wide range of tumor cells [48]. PCa cells induce senescence in a subset of immunosuppressive neutrophils by secreting apolipoprotein E (APOE), which binds to the Triggering receptor expressed in myeloid cells 2 (TREM2). Senescence arrest cell proliferation in a stable manner but cells remain metabolically active, resist apoptosis and induce tolerogenic state in T cells. APOE belongs to a family of lipid-binding proteins that serve as major transporters of systemic cholesterol and triglycerides. The APOE/TREM2 axis correlates with a poor prognosis in PCa patients. Specifically eliminating senescent immunosuppressive neutrophils using histone deacetylase inhibitors enhances the effectiveness of standard therapeutic approaches [50].

### 4.2. Growth Factor Receptors Reside Inside Lipid Rafts

In the context of PCa, elevated cholesterol levels promote disease progression by inducing the epithelial-to-mesenchymal transition (EMT) through the activation of the extracellular-regulated protein kinases 1/2 (ERK1/2) pathway, mediated by the EGFR, and the accumulation of adipocyte plasma membrane-associated proteins (APMAPs) in LRs. Notably, high cholesterol levels in LRs inhibit EGFR internalization. Consistently, both APMAP mRNA and protein levels are elevated in clinical PCa samples [51]. HER2, also called EGFR 2, has been detected on the cell surface of quiescent (G0) PCa cells, which often exhibit resistance to chemotherapy and are associated with recurrence following dormancy [52].

Another member of the EGFR family, HER3, has been found to be overexpressed in human CRPC samples, correlating with enhanced proliferation and survival. Notably, these effects can be counteracted by a neutralizing anti-neuregulin 1 (NRG1), which serves as the ligand for HER3 [53]. NRG1 is often generated by fibroblasts. In addition to these receptors, G protein-coupled receptors (GPCRs), particularly the G protein-coupled estrogen receptor (GPER), are detected in the cytoplasm of basal epithelial cells, as well as in Gleason patterns 2 or 3, but not in luminal secretory epithelial cells. Various mechanisms associated with GPCRs have been reported in PCa, either alone or in conjunction with estrogen receptors (ERs) [54,55,56].

Similarly, lipid rafts (LRs) are significantly enriched in cancer stem cells (CSCs) compared to non-stem cancer cells, and key CSC markers, such as CD24, CD44, and CD133, are located within these lipid rafts, underscoring their pivotal role in CSCs. LRs play a crucial function in facilitating CSC self-renewal, the EMT, drug resistance, and the CSC niche [57,58]. In the context of PCa, CSCs exhibit heightened expression levels of CD44 and CD133 [59]. In vitro assays have demonstrated that cholesterol depletion disrupts lipid rafts, inhibits cancer cell migration, and abolishes β-catenin activation and the CSC phenotype [58], emphasizing the involvement of cholesterol in regulating phenotypic plasticity.

### 4.3. Interaction of Cholesterol with Androgen Receptor Signaling, the Critical Route of Prostate Cancer

AR expression has been detected in nearly all primary and metastatic PCa cases, regardless of stage or grade, and is sustained in the majority of androgen-independent PCa and CRPC. Intense nuclear AR staining in CRPC bone metastases is associated with a worse clinical outcome [60,61]. Some CRPC cases that emerge in patients are linked to the hyperactivation of the androgen signaling pathway due to various factors, including AR gene amplification, AR mutations, the expression of constitutively active AR splice variants (with AR-V7 being the most common variant found in 75% of metastatic PCa cases), intratumoral androgen synthesis, the altered expression and function of AR coactivators, and the signaling crosstalk with other oncogenic pathways [1,62,63]. AR mutations have been identified in 60% of metastatic tumors [64,65] and 1% of primary PCa cases [65]. Functionally, these mutations enable antiandrogens to act as AR agonists and activate AR through estrogens, progesterone, dehydroepiandrosterone (DHEA), androstenediol, and glucocorticoids [66,67,68]. Studies in both patients and animal models have demonstrated that certain point mutations convert AR antagonists into potent agonists [69]. AR-V7 is an aberrantly spliced mRNA isoform of AR, leading to a protein lacking the C-terminal ligand-binding domain but retaining the transcriptionally active *N*-terminal domain. Despite its inability to bind ligands, AR-V7 remains constitutively active in a ligand-independent manner and can drive the growth of CRPC [62]. Also, APUC genes have been described as important predictors of clinical response in CRPC, belonging to a family characterized by androgen production, uptake, and conversion, based on genomic analyses of patient germlines [70].

Cholesterol derived from lipid rafts in PCa cell lines has been demonstrated to interact with the *N*-terminal domain of testosterone-activated AR and form complexes with transient receptor potential melastatin 8 (TRPM8). This interaction inhibits the function of TRPM8, a non-selective cation channel involved in the calcium signaling of tumor and stromal cells and has been proposed as a molecular target in cancer development and progression [71,72,73]. This interaction significantly enhances the migratory capacity of PCa cells, promoting invasion and metastasis. Activated AR has a dose-dependent regulatory effect on the function of the TRPM8 channel [74], in addition to its known ability to induce the transcription of TRPM8 through binding to androgen response elements (AREs) [75]. Likewise, prostate-specific antigen (PSA) has also been observed to modulate TRPM8, facilitating its accumulation in the plasma membrane, similar to the function described for AR but mediated by a G protein-coupled membrane receptor and AKT signaling associated with activated AR [76,77]. However, various findings suggest that additional factors might modulate the cell response to the TRPM8 channel [72]. It is plausible that these factors collectively influence the TRPM8 channel response in PCa [73,78].

SREBP1 has been implicated in PCa cell proliferation, migration, and invasion. Studies have revealed that an AR/mTOR complex promotes SREBF1 expression and activity, primarily due to the constitutive activation of mTOR resulting from the loss of function of PTEN [79]. Moreover, the AR-SREBP1 axis has been identified, forming a self-regulating interaction between the activation of SCAP by AR and SREBP1, which subsequently increases the expression of AR [80,81]. Androgens are recognized as key activators of SREBPs under both physiological conditions and in steroid-regulated cancers [82]. One form of regulation occurs through the stimulation of SCAP expression [83], leading to a switch in the isoform expression of INSIG [84] and the upregulation of several enzymes involved in fatty acid and cholesterol synthesis under the transcriptional control of AR [13]. Perturbations in cholesterol metabolism have been predominantly identified in high-grade tumors [3]. Correspondingly, the inhibition of fatty acid synthase (FASN) enhances the immune response to castration-resistant PCa cell line tumors and decreases the expression and transcriptional activity of AR and the AR-variant splicing 7, resulting in a better response to hormone treatment [85]. The secretion of cholesterol into the intracellular space is regulated by ATP-binding cassette (ABC) transporters, and data have shown the tissue-dependent regulation of ABC transporters by estrogens, progesterone, and androgens [86].

### 4.4. The Androgen Receptor–Microbiome–Diet Axis in Prostate Cancer

The gut microbiome is linked with diet; also, the immune response is connected to the modulation of immune function, resulting in a different response to immune checkpoint therapy and serving as a source of testosterone. Men with CRPC have an increased abundance of gut bacteria with androgenic functions. Men with high-risk PCa share a specific gut microbial profile. Most importantly, a high-fat diet causes gut dysbiosis and gut bacterial metabolites such as short-chain fatty acids, propionate, and acetate, which enter systemic circulation, resulting in the promotion of PCa growth; this is called the “gut-prostate axis” [87,88]. Humans can utilize absorbed acetate and propionate as substrates for lipid, glucose, and cholesterol metabolism [89]. Androgen deprivation in mice and humans promotes the expansion of defined commensal microbiota capable of converting androgen precursors into active androgens. The depletion of these microbiota and their replacement with Prevotella stercorea control the growth of PCa [90]. Clostridium scindens from fecal human microbiota can convert glucocorticoids into androgens [91], and similarly, Ruminococcus gnavus metabolize pregnenolone to androgenic steroids [90].

In 2016, the International Agency for Research on Cancer (IARC) recommended the avoidance of weight gain based on a body of evidence from around 1000 studies linking obesity to at least 13 types of cancer. This correlation is often linked to higher levels of lipids in the bloodstream [92]. Obesity and altered lipids are associated with the induction of vasculature formation and inflammation through the ETS transcription factor ERG, a gene that encodes key regulators of embryonic development, cell proliferation, angiogenesis, inflammation, and apoptosis. In PCa, the protein ERG contains an ETS DNA-binding domain and a PNT (pointed) domain which is implicated in the self-association of chimeric oncoproteins like TMPSSR2-ERG and NDRG1-ERG [3,93,94]. Individual metabolites correlate with Gleason scores and impact the translocation of ERG, highlighting altered fatty acid oxidation. Specifically, cholesterol has been shown to be negatively correlated with Gleason score [3]. In fact, metabolomic differences between Gleason 4 + 3 and Gleason 3 + 4 have been described, suggesting that lipids could be considered markers of PCa aggression [95], which is related to the identification of metabolic signatures of PCa [3,5], similar to other new personalized signatures developed in early PCa based on multimodal biomarkers [96].

### 4.5. The Role of the cGAS–Sting Pathway in Prostate Cancer

The cGAS–STING pathway plays an important role in the immune defense against both infections and cancer. Upon the activation of the STING protein, various signaling cascades are initiated, ultimately leading to the activation of interferon regulatory factor 3 (IRF3), nuclear factor-kappa B (NF-κB), and the expression of interferon (IFN) and autophagy [97]. Acting as a vital component of the genomic stability system within the cytosol and endolysosomal compartments, the cyclic GMP-AMP synthase (cGAS) detects double-stranded DNA (dsDNA) and functions as an adenosine monophosphate (AMP) synthase [98].

In the context of PCa, which is often characterized as immunologically “cold” due to limited responses to checkpoint inhibitory therapy, the significance of the STING pathway is underscored. STING activation leads to the upregulation of genes associated with antigen presentation, Th1 chemokine signaling, interferon response, and the expression of programmed cell death protein 1 (PD-L1). These functions of the STING pathway imply its potential relevance in augmenting the immunogenicity of PCa and enhancing the efficacy of immune-based therapies. Consistent with these observations, the enhanced expression of enhancer of zeste homolog 2 (EZH2), a negative regulator of the STING pathway, has been linked to the heightened intratumoral trafficking of activated CD8+ lymphocytes (LT), an increase in M1 tumor-associated macrophages (TAMs), and the reversal of resistance to PD-1 checkpoint therapy [99]. These findings are in consonance with Speckle-type POZ protein (SPOP), another modulator of dsDNA, which is mutated in 15% of PCa patients. SPOP is crucial for regulating the balance between immunosuppressive and antitumor activities downstream of STING. It can shift the pathway toward antitumor cGAS–STING–IFN-β signaling [100]. Also, in vivo assays suggest that the MVA pathway and IFN signaling pathway are part of a metabolic circuit, sensing each other [101].

Recent research efforts have focused on utilizing the cGAS–STING pathway as a therapeutic target for the treatment of PCa, and it has been demonstrated that ionizing radiation can activate this pathway and enhance the response of CD8+ lymphocytes [102,103]. On the other hand, the cGAS–STING pathway has also been linked to promoting tumor proliferation. Propionibacterium acnes, a common microorganism in normal prostate tissues and PCa, can activate the cGAS–STING pathway and induce the expression of interferons, which subsequently promotes the growth of PCa [98].

### 4.6. Function of Stroma Elements in Prostate Cancer

Cancer-associated fibroblasts (CAFs) represent the predominant stromal cell type in the tumor microenvironment (TME) [104,105], and their abundance is notably pronounced in PCa [106]. These cells, primarily derived from tissue-resident fibroblasts and other local cells, including tumoral epithelial PCa cells [104,105,107,108,109,110], exert a significant influence on tumorigenesis. Notably, only grafts containing initiated prostate epithelium and CAFs generate tumors [111,112]. CAFs induce angiogenesis, immunosuppression, metastatic progression, and resistance to therapy. However, in certain contexts, CAFs have been linked to exerting antitumoral activity during the early stages of tumorigenesis [113]. This finding aligns with recent research indicating rapid epigenetic remodeling within hours of tumor-specific CD8+ T lymphocytes after sustained exposure to tumor antigens, resulting in a persistent dysfunctional state despite robust activation and proliferation [114].

New evidence suggests that tumor cells can circumvent the constraints posed by androgen deprivation therapy (ADT) through the acquisition of glucosamine from CAFs. This process leads to an elevation in O-GlcNAc levels and, ultimately, triggers the induction of 3β-Hydroxysteroid dehydrogenase-1 (3βHSD1), the crucial enzyme responsible for the conversion of dehydroepiandrosterone (DHEA) to the highly potent androgen dihydrotestosterone (DHT) [115]. Additionally, mutations occurring in the AR’s ligand-binding domain cause the expanded responsiveness of the AR signaling pathway to nonandrogenic steroid molecules and antiandrogens, along with the expression of constitutively active AR splice variants, alterations in the function of AR coactivators, and intricate crosstalk with other oncogenic pathways [1,116,117]. In this context, cholesterol plays a role in regulating the expression of AR cofactors. Elevated levels of SRC-1, SRC-2, SRC-3, and PCAF gene and protein expression are observed concurrently with heightened AR gene and protein expression. This interplay exerts a notable influence on the initiation and advancement of PCa in a murine model of CRPC [117]. In agreement with this, the complexity of the AR is further highlighted by the observation that CRPC is associated with AR overexpression due to clonal selective pressure in the androgen-depleted environment, leading to gene upregulation. Notably, androgen-independent cells require 80% less androgens for growth [118], and approximately 30% of these cells exhibit high-level amplifications [119]. Furthermore, AR mutations have been identified to increase sensitivity to androgens by four orders of magnitude lower than that required for androgen-dependent PCa cells [120]. This hyperactivation of the androgen signaling pathway leads to CRPC.

It has been observed that, in PCa, there is a bidirectional relationship between the signaling pathway of AR and the TAMs. AR activation has been described as essential for the ability to induce tumor cell death by TAMs, which is why ADT would be counterproductive to the local innate immune response [121]. However, when TAMs become pro-tumor, they can transfer cholesterol to tumor cells in vitro by increasing androgen production and AR activation, suggesting the need to limit cholesterol transfer, the MVA pathway, or TAMs as adjuvant therapy in the context of PCa [122].

### 4.7. Mitochondria in the Metabolic Deregulation of Prostate Cancer

**Figure 2:**
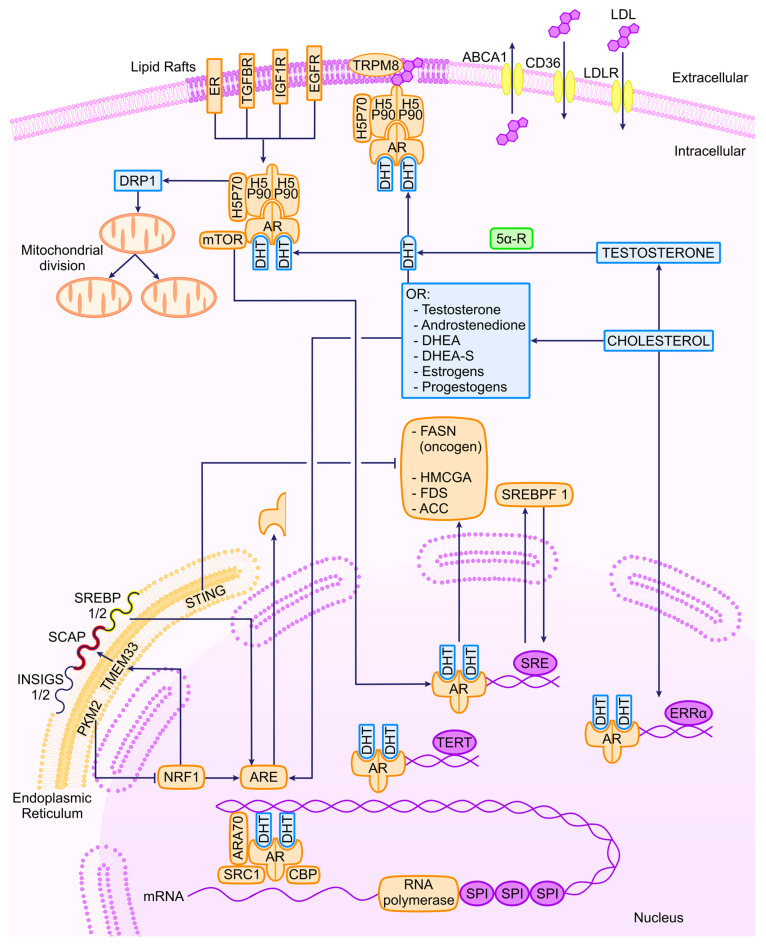
Transcriptional regulators of AR and related molecules in PCa. ER, estrogen receptor; TGFBR, tumor growth factor β receptor; IGF1R, insulin-like growth factor 1 receptor; EGFR, epithelial growth factor receptor; TRPM8, transient receptor potential cation channel subfamily M (melastatin) member 8; ABCA1, ATP-binding cassette subfamily A member 1; LDLR, LDL cholesterol receptor; DRP1, dynamin-related protein; DHT, dihydrotestosterone; 5α-R, 5 alpha receptor; DHEA, dehydroepyandrosterone; PTEN, phosphatase and tensin homolog; cGAS, cyclic GMP-AMP synthase; PKM2, pyruvate kinase M2; TMEM33, transmembrane protein 33; STING, stimulator of interferon response cGAMP interactor 1; NRF1, nuclear respiratory factor 1; ARE, androgen response element.

Metabolic alterations induced by oncogenesis in PCa are sustained through mitochondrial activity and the involvement of dynamin-related protein 1 (DRP1), a mediator of mitochondrial fission. Elevated DRP1 levels in both androgen-sensitive and CRPC cell lines are associated with AR signaling, leading to an enhancement of pyruvate transport into the mitochondria. This, in turn, promotes oxidative phosphorylation (OXPHOS) and lipogenesis, which are hallmark characteristics of PCa [1]. Similarly, the upregulation of the pyruvate kinase M2 isoform (PKM2) is critical for controlling lipid homeostasis. The regulation of nuclear respiratory factor 1 (NRF1), responsible for the transcription of the endoplasmic reticulum (ER) transmembrane protein 33 (TMEM33), is a key element of this mechanism. As mentioned earlier, the SREBP proteins are localized in the ER, and TMEM33 recruits the RNF5 enzyme to facilitate SCAP degradation and activate the MVA pathway. These findings are associated with the observation that PKM2 is predominantly expressed in PCa cells as opposed to normal prostate cells. The downregulation of PKM2 is linked to a reduction in tumor growth, while the global knockout of PKM2 accelerates the growth of allografted tumors [123]. A part of these considerations is shown in a schematic way in Figure 2.

### 4.8. The Telomerase Complex Is Related to Androgen Receptor Signaling

Telomeres are the DNA-protein structures that cap the ends of linear chromosomes and protect them from fusing end to end [124]. The telomerase is a protein complex (HSP90, hTERC, diskerin, TEP1, p53, hTERT), the canonical function of which is to protect against telomere shortening [125]. The non-canonical functions of the telomerase contribute to cancer progression through apoptosis resistance; an increase in mitochondrial membrane potential and a decrease in ROS; gene expression; and signal transduction [125].

In PCa cells, telomere stability may be modulated via an allosteric mechanism in-volving the AR. The AR interacts with telomeric proteins, specifically TIN2 from the shelterin complex, and exhibits the capability to disrupt these interactions in AR-positive PCa cell lines when treated with the AR antagonist bicalutamide [124]. Additionally, there is evidence suggesting a concentration-dependent impact of androgens on human telomerase reverse transcriptase (hTERT) expression. Under physiological androgen levels, characterized by low androgen levels, hTERT expression is induced. Conversely, at high supraphysiological androgen levels, hTERT expression is suppressed by the inhibitor of growth 1 (ING1) and 2 (ING2) [126]. Recently, it has been postulated through bioinformatic studies that the AR, zinc finger proteins, and telomeres modulate the global gene expression pattern during PCa progression [127]. Conflicting findings about the correlation of telomerase expression and clinicopathological indicators may be consistent with noise caused during processing of tumor tissues by surrounding normal tissue expressing little or no telomerase activity. [128,129].

The cholesterol-derived hormone estrogen, specifically 17β-estradiol (E2), has the capacity to stimulate the transcription of the human telomerase reverse transcriptase (hTERT) gene in PCa cells. This induction results in an increase in telomerase activity within a short period. Notably, estrogen receptors (ERs) collocate with the hTERT promoter in PCa cells, indicating a direct interaction between estrogen signaling and the regulation of telomerase activity in PCa [130]. The significance of these findings lies in the association of estrogens with dihydrotestosterone biosynthesis through the “backdoor pathway.” This pathway, which is not only implicated in the human testis and adrenal glands but also in the classical pathway for androgen synthesis, has been linked to tumor resistance to castration [131].

## 5. Statins as Adjuvant Therapy in Prostate Cancer

Statins, widely used for reducing the de novo production of cholesterol in the blood, were first developed from Penicillium citrinum in 1976 [132]. They exert their effect by competitively inhibiting HMG-CR (see Figure 1), leading to reductions in serum total cholesterol, low-density lipoprotein (LDL) cholesterol, apolipoprotein-B (Apo-B), and triglycerides, thereby aiding in the prevention of cardiovascular disease (CVD) [133]. Their remarkably high affinity for HMG-CR, approximately 1000 times higher than the endogenous substrate, results in the induction of LDL cholesterol receptors and subsequent reductions in blood levels of this type of cholesterol. Some statins, such as simvastatin, lovastatin, and pravastatin, are derived from fungal sources, while others are synthetic compounds [7].

The ability of statins to produce intracellular effects is closely linked to their chemical structure, with lipophilic statins having a higher capacity for diffusion into the intracellular space [134,135]. This heightened intracellular diffusion capability is associated with their ability to induce apoptosis [136] and may also contribute to certain adverse effects [137]. Statins play a critical role in modulating cholesterol levels in lipid raft-dependent functions within cancer cells and tumors. In the context of PCa cell lines, the increase in intracellular cholesterol in response to the activation of PI3K can be countered by the administration of statins [138,139,140].

In vitro and in vivo experiments indicate that statins can decrease cellular plasticity by promoting a mesenchymal-like cell state. This transition enhances the ability of metastatic seeding on the one hand but suppresses the reorganization of cells into secondary tumors on the other. In essence, the influence of statins leads to an augmentation of the EMT while inhibiting the vital and dynamic reversal in cellular phenotype known as the mesenchymal-to-epithelial transition (MET), which is crucial for the colonization of distant organs. This overall process, termed epithelial-mesenchymal plasticity (EMP), characterized by a combination of cell states displaying both partial epithelial and mesenchymal characteristics, is notably curbed. In the case of PCa, the utilization of rosuvastatin has shown a reduction in cellular plasticity [141,142], whereas lovastatin or simvastatin has been linked to the deactivation of RhoA, triggering cancer cell apoptosis and inducing cell cycle arrest in the G1 phase [143].

Bisphosphonates are other compounds that act on the MVA pathway; however, they inhibit farnesyl-pyrophosphate synthase, an enzyme downstream HMGCR in this pathway [144]. Bisphophonates can bind to mineral bone surfaces [145] and are used in oncology for the management of skeletal complications and/or bone metastases. In addition, some bisphosphonates have been shown to exhibit direct anticancer properties [146,147], indicating that targeting the MVA pathway could have beneficial effects, at least in certain cancer types affecting bone tissue.

### 5.1. Adverse Effects Induced by Statins

The detrimental effects associated with high-dose statin use, known as statin-associated side effects (SASs), lead to approximately 50% of patients discontinuing treatment within a year [148,149,150]. The methylation-induced downregulation of ABCG1 due to statins has been linked to an increased risk of developing type 2 diabetes [151]. Various studies suggest that 9–12% of statin recipients face an elevated risk of type 2 diabetes [152]. However, a recent meta-analysis has indicated that the actual increase in risk equates to one case per 100–200 patients treated [153]. Despite these potential side effects, the benefits of statin use outweigh the adverse outcomes, making it highly recommended for individuals with cardiovascular risk.

Furthermore, other less explored symptoms include neurological, neurocognitive, hepatotoxicity, nephrotoxicity, gastrointestinal, urological–genital, and reproductive effects. Statins disrupt the Akt pathway, leading to mitochondrial dysfunction, resulting in insufficient energy production for highly energy-demanding tissues such as muscles. This gives rise to what is known as statin-associated mitochondrial symptoms (SAMSs). SAMSs are the primary reason for treatment discontinuation, and they can manifest as weakness, muscle pain, fatigue, myalgia, tendon pain, night muscle cramps, and, in extreme cases, rhabdomyolysis [154,155]. These adverse effects are closely linked to several key mechanisms. Firstly, the reduction in circulating lipoproteins, which transport a substantial portion (74%) of CoQ10, leads to the depletion of CoQ10 [155,156]. CoQ10 plays an essential role in the functioning of mitochondrial electrical complexes. Consequently, this depletion contributes to mitochondrial depletion and triggers oxidative stress. These effects are further compounded by the downregulation of peroxisome proliferator-activated gamma coactivator receptor (PGC)-1α and PGC-1β [157].

Moreover, the inhibition of the electron transport chain and the disruption of Ca2+ metabolism through increased mitochondrial Ca2+ efflux via the Na+-Ca2+ exchanger [158] and, secondarily, the exit of Ca2+ from the sarcoplasmic reticulum mediated by ryanodine receptors [159] result in the activation of calpain. Calpain is responsible for the translocation of BAX to the outer mitochondrial membrane, leading to the release of cytochrome C into the cytosol and the subsequent activation of the intrinsic pathway of apoptosis [160].

### 5.2. Clinical Evidence of the Antitumoral Effects of Statins

The mevalonate pathway has been found to play a crucial role in the development of PCa by producing molecules that are either directly or indirectly related to the function of AR, which is the molecule at the center of this pathology. Cholesterol precedes the generation of steroid hormones in the mevalonate pathway and has been found to have a very relevant structural role in the formation of LRs, which are the fundamental axis of the cell’s functioning because they coin the receptors that are the starting point for a cellular response to an external stimulus. Cholesterol directly or indirectly regulates several signaling pathways that are interconnected with the AR signaling pathway.

Clinical data about the use of statins as antitumoral agents are contradictory, but the level of evidence of this contribution must be evaluated before arriving to any solid conclusions.

In 2018, Murtola and colleagues detected a 14% reduction in the tumor proliferation index Ki-67 in patient samples after a 28-day intervention with 80 mg of atorvastatin [161]. A subsequent study demonstrated the positive impact of 80 mg of fluvastatin on cell proliferation, with detectable concentrations in prostate tissue and increased apoptosis in 33 patients who had undergone prostatectomy. Fluvastatin, after 4–12 weeks of use, attained adequate blood levels consistent with histopathological evidence supporting the antitumor role of statins [162,163]. Although these data manage to harmonize the contribution of statins at the cellular level with consistent pharmacological parameters, due to the small sample size, analyses of these results remain inconclusive, emphasizing the need to establish a more robust connection between fundamental scientific evidence and clinical applications [164].

Prognostic indicators are needed to help urologists establish the most appropriate therapy for PCa patients, and we have previously mentioned some of them, like hTERT expression determined in prostate tissue, which is positively correlated with various clinicopathological features, such as Gleason score, tumor differentiation, serum levels of PSA, recurrence and worse survival following radical prostatectomy [128,129]. Patients with tumors with little infiltration of TAMs and CAFs exhibit significantly improved recurrence-free survival (RFS) rates and more favorable outcomes from hormonal therapy compared to those with high infiltration levels. In addition, TAMs and CAFs correlate with serum PSA levels, Gleason score and T stage [165]. CAFs have also been proposed as PCa markers. It has been observed that both HMGCS2 and aldoketo reductase 1 member C3 (AKR1C3) increase their levels, particularly in CRPC cell lines, in cocultures with CAFs. Therapy with simvastatin and AKR1C3 inhibitors decreased the growth of PCa lines, and this effect also extended to CRPC lines and enzalutamide resistant [166,167]. These enzymes showed a significant elevation and are correlated with the Gleason score in human PCa specimens [168].

However, a limitation has been identified in preclinical studies on the anticancer efficacy of statins: the need to achieve concentrations in the micromolar range to effectively inhibit cell proliferation. These concentrations are currently unattainable in clinical practice without inducing significant side effects [169]. Our approach involves the use of statins as a complement to other well-established treatments in PCa and not as a single therapy to inhibit cell proliferation. To reduce the availability of tumor cells for immune escape through the activation of the RA signaling pathway, it is crucial to reach therapeutic levels similar to those required to prevent cardiovascular disease [170].

Dickerman and colleagues emphasize the significance of proper randomization in understanding discrepancies. They found that statin therapy did not have a significant impact on the incidence of PCa in an emulating study that analyzed the electronic health records of 733,804 adults over a decade [171]. They did not analyze the potential benefits of using statins to mitigate the severity of PCa. However, this study does not diminish the strong evidence that has been generated from well-randomized prospective trials that have shown positive results related to combining statins with other therapies.

An initial analysis from the REDUCE database revealed that the use of statins did not increase or diminish the development of PCa [172]. However, five years later, the accumulated data revealed that high total serum cholesterol and high HDL cholesterol were associated with an increased risk of high-grade PCa [173]. In accordance with this, a meta-analysis found that statin use was associated with a 20% lower risk of advanced PCa based on data from 15 cohort and 12 case–control studies [174] and findings from two large prospective studies with substantial follow-up support, the Health Professionals Follow-up Study (24 years) and the Atherosclerosis Risk in Communities (ARIC) study (20 years) [175,176]. In the same way, recent robust findings show that statins could be useful in the advanced stages of PCa when combined with AR axis-targeted therapies (ARATs) for men with high-risk hormone-sensitive PCa (HSPC) or CRPC after radiotherapy. This prospective analysis, which took place over almost 7 years, determined that, in two months, statins improved overall survival (OS) and PCa-specific survival [177].

These substantial findings support other less consistent findings, like thos derived from a post hoc analysis in patients previously treated with docetaxel [178] or a meta-analysis from three phase III studies evaluating enzalutamide: AFFIRM, PREVAIL, and PROSPER; a multivariate analysis suggested that statins are associated with improved OS, but significant limitations meant that the results were confounded by the baseline cardiovascular risk and original indication of statins [179]. These findings agree with those of other studies, specifically one in which a 27% reduction in the risk of overall mortality and a 35% lower risk of specific mortality were established in statin users among men with PCa receiving ADT or ARATs in 25 cohorts; however, these studies had great heterogeneity, which prevented an adequate comparison, despite studies of quality, sensitivity, and strategies to reduce biases [180].

An analysis of a Finnish cohort of 14,424 men with PCa conducted over the course of 18 years reported a decreased risk of starting androgen deprivation therapy (ADT) and PCa-related death in patients who initiated statin use 5 years before diagnosis [181]. Similarly, a large population-based electronic database in the United Kingdom was used to evaluate 11,772 patients over 4.4 years, and it was found that a decreased risk of PCa-specific mortality is associated with statin use, with a stronger association being observed when statins were used before diagnosis [182].

The secondary role of statins in the prevention of PCa was examined in a systematic meta-analysis which included prospective, retrospective, and randomized controlled trials. This study found that the combination of statin use and androgen deprivation therapy (ADT) was associated with a significant reduction in the risks of mortality of all-causes and PCa-specific mortality. Similarly, the combination of statins and radical prostatectomy improved cancer-specific mortality. However, the authors admitted that differences in the time, dose, and duration of statin treatment caused heterogeneity, and the most effective type of statin was not established [183].

One study that did not achieve findings that support the use of statins as a complementary therapy in PCa, despite involving prospective, randomized, and double-blind trials, is the PRO-STAT, a study that involved 181 patients and the use of suboptimal doses of atorvastatin (20 mg/day) after RP [184]. However, this study faced criticism due to the relatively low doses of atorvastatin used, which were not effective in sufficiently reducing cholesterol levels [162]. A cohort study about the relation between statin use and the aggressiveness of PCa (higher Gleason score) established that the benefits are dose-dependent, meaning that the use of statins may continue for a relatively longer time (>11 months) [185].

Certainly, some strong evidence provided by extensive prospective studies supports the use of statins due to their antitumor properties and well-underlined benefits when used in combination with RP, ADT, and ARATs. In addition to clinical data, in vitro results have highlighted the diverse mechanisms linking the MVA pathway and other metabolic pathways to the AR pathway and PCa. These mechanisms encompass castration resistance, metastasis, and the response to different therapeutic approaches. These intricate processes not only involve the epithelial components but also underscore the critical role of stromal elements and microbiota in the progression of PCa, and recently, this has been supported by congruent evidence from metabolomic studies.
